# Synchronous hydatid cysts of the pancreas and liver: A rare double localization

**DOI:** 10.1016/j.idcr.2026.e02532

**Published:** 2026-02-19

**Authors:** Hazem Beji, Yassine Kallel, Houda Gazzah, Aymen Laaribi, Mohamed Mongi Mighri, Hassen Touinsi

**Affiliations:** a University of Tunis El Manar, Tunisia; b Department of General Surgery, University Hospital Mohamed Taher Maamouri, Nabeul, Tunisia

**Keywords:** Hydatid cyst, Pancreas, Liver, Parasitology, Surgery

A rural 62-year-old woman presented with epigastralgia for ten months. Physical examination and laboratory studies were normal. Abdominal computed tomography (CT) scan showed a unilocular cystic lesion in the right hepatic lobe. It showed also a unilocular, well-organized, cystic lesion, with a partially calcified wall, of the body and tail of the pancreas (Fig. 1). Hydatid serology using the ELISA test was positive.

**Fig. 1:**
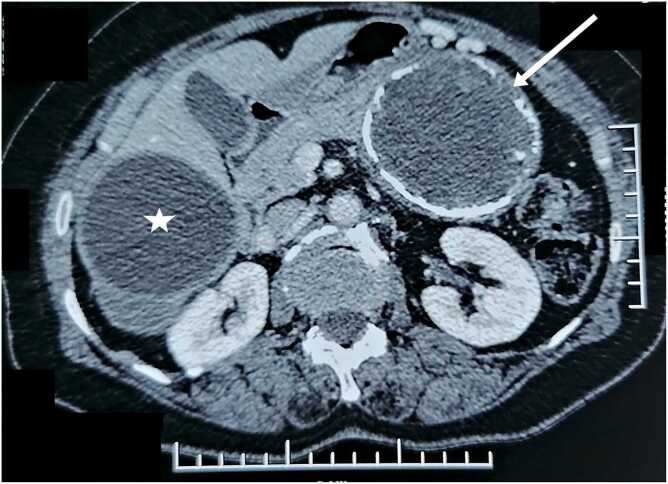
Abdominal CT scan showing the hydatid cysts of the pancreas (arrow) and the liver (star).

A bilateral subcostal incision was performed. A partial cystectomy was performed for the hydatid cyst of the liver. For the pancreatic cyst, we performed total cystectomy and drainage regarding the body-tail region of the pancreas (Fig. 2, Fig. 3). The postoperative course was uneventful. Pathology confirmed the diagnosis of the liver and pancreas Hydatid cyst.

**Fig. 2:**
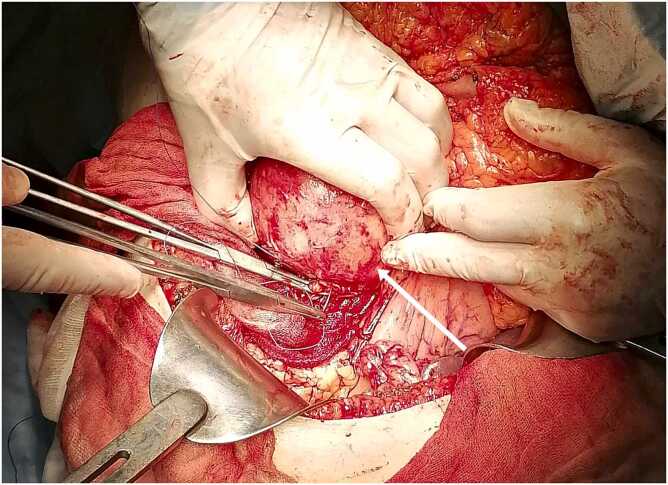
Intraoperative view demonstrating the pancreatic hydatid cyst (arrow).

**Fig. 3:**
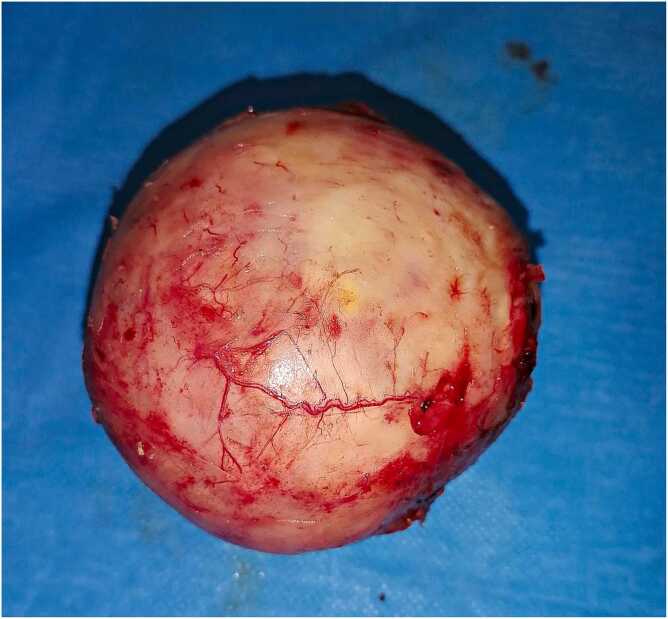
Surgical specimen (the pancreatic hydatid cyst).

Pancreatic Hydatid cyst (PHC) is a rare situation, representing less than 1 % of all the localizations of the hydatid cyst [1]. Patients remain asymptomatic for long periods. PHC can be discovered at the stage of complications such as jaundice, cholangitis, and pancreatitis [2].

Preoperative diagnosis is difficult. Differential diagnoses such as pancreatic cystadenoma, pseudocyst, and neuroendocrine tumor can be evoked [3].

The only curative treatment is surgery. Radical surgery, when feasible, is the best option. It permits to avoid recurrence [4].

## CRediT authorship contribution statement

**Aymen Laaribi:** Writing – original draft. **Mohamed Mongi Mighri:** Validation, Writing – review & editing. **Yassine Kallel:** Investigation, Methodology, Resources, Software. **Houda Gazzah:** Data curation, Investigation. **Hassen Touinsi:** Writing – review & editing. **Hazem Beji:** Conceptualization, Investigation, Methodology, Project administration, Writing – original draft.

## Ethical approval

Written informed consent was obtained from the patient for the publication of this case report and the accompanying images.

## Funding source

This research did not receive any specific grant from funding agencies in the public, commercial, or not-for-profit sectors.

## Declaration of Competing Interest

The authors declare that they have no known competing financial interests or personal relationships that could have appeared to influence the work reported in this paper.
